# Van Der Waals Ferroionic CuInP_2_S_6_: Emergent Properties and Device Application

**DOI:** 10.3390/ma19081586

**Published:** 2026-04-15

**Authors:** Muzhi Li, Zhuoyin Peng, Dongdong Zhang, Xueyun Wang, Weiyou Yang, Zhao Liang, Xingan Jiang

**Affiliations:** 1 School of Energy and Power Engineering, Changsha University of Science & Technology, Changsha 410014, China; 23206031323@stu.csust.edu.cn; 2 Institute of Micro/Nano Materials and Devices, Ningbo University of Technology, Ningbo 315211, China; dongdongzhang@mail.sdu.edu.cn (D.Z.); weiyouyang@tsinghua.org.cn (W.Y.); 3 School of Aerospace Engineering, Beijing Institute of Technology, Beijing 100081, China; xueyun@bit.edu.cn

**Keywords:** van der Waals ferroelectrics, CuInP_2_S_6_, polarization, ferroionic properties, ion migration

## Abstract

Low-dimensional van der Waals (vdW) ferroelectrics are promising for next-generation low-power, non-volatile electronics and brain-inspired computing. Among them, CuInP_2_S_6_ (CIPS) has emerged as one of the most intensively explored systems. Distinct from conventional ferroelectrics, CIPS features a strong “ferroionic” coupling between ferroelectric order and long-range Cu^+^ migration, unlocking unique properties such as multiple polarization states, negative capacitance, and richly tunable conductance states. To date, however, a comprehensive review centered on this ferroionic coupling remains lacking. This review aims to fill that gap by systematically elucidating the ferroionic coupling mechanism, summarizing its manipulation through chemical composition engineering and external fields, and clarifying the dynamic conductive responses and related mechanism. This review further surveys the high-performance CIPS-based nanoelectronic devices enabled by unique properties and concludes with an outlook on future challenges and research directions.

## 1. Introduction

The emergence of two-dimensional van der Waals (vdW) ferroelectric materials has opened exciting avenues for next-generation low-power, non-volatile memory, logic, and brain-inspired computing [1,2,3,4,5,6,7,8,9,10,11,12,13]. Their atomically thin nature, clean interfaces, and compatibility with vdW heterostructure integration offer distinct advantages over traditional ferroelectrics. Among the expanding family of vdW ferroelectrics, copper indium thiophosphate, CuInP_2_S_6_ (CIPS), has emerged as one of the most intensively studied and functionally rich systems, distinguishing itself not merely by the presence of ferroelectricity but by the unique nature of its origin.

Recent years have witnessed rapid advances in the CIPS ferroionic system, unveiling a host of novel phenomena [14,15,16,17,18,19,20,21,22,23,24,25,26,27,28]. These include the existence of multiple, electrically accessible polarization states (low-polarization LP and high-polarization HP) forming a quadruple-well energy landscape [29,30,31,32,33,34], the emergence of negative capacitance driven by ionic kinetics [31,35,36], the formation of topological polar textures like skyrmion-like bubbles [37,38], and highly tunable conductance states that bridge memristive and neuromorphic functionalities [15,21,24,26,39,40,41,42,43,44,45,46,47]. These diverse and emergent properties are not isolated discoveries but are fundamentally interconnected. They share a common physical root: a strong, intrinsic coupling between the ferroelectric order and long-range Cu^+^ migration—a phenomenon termed “ferroionic” coupling. Crucially, these ferroionic couplings are highly responsive to chemical composition [16,48,49] or dynamic external fields [19,23,26,50],which enables dynamically engineered polarization dynamics and conductance states.

It is worth noting that recent years have also witnessed progress in other analogous variants, such as AgInP_2_S_6_ [1,2,3,4,5,6,7,8,9,10,11,12,13] and CuInP_2_Se_6_ [1,2,3,4,5,6,7,8,9,10,11,12,13]. These materials exhibit a robust high-speed photoresponse and superior optical performance compared to CIPS. However, their ferroelectric properties are significantly diminished, failing to realize remarkable ferroionic dynamics and related emergent properties. In this review, we focus on CIPS primarily because the central theme is ferroionic properties and their associated emergent phenomena and device applications.

To date, significant progress has been achieved in ferroionic CIPS, however, a comprehensive review centered on this ferroionic coupling remains lacking. There is an urgent need for a holistic framework that traces the ferroionic coupling from its microscopic origins to the emergent properties and device applications it enables. This review provides a comprehensive framework to understand this versatile ferroionic material. The core sections delve into the mechanisms of ferroionic coupling and its modulation by chemical composition or various external fields, which forms the basis for dynamic control. The complex conductive responses and related mechanisms are discussed. Finally, we showcase how these unique ferroionic characteristics are being harnessed in pioneering device concepts for non-volatile memory, reconfigurable electronics, and neuromorphic computing, concluding with an outlook on persistent challenges and promising research directions.

## 2. The Crystal Structure and Ferroelectricity

As a prototypical two-dimensional vdW ferroelectric, CIPS derives its ferroelectricity from its unique crystal structure and the site occupancy of Cu ions within it. Understanding the origin of this ferroelectricity and how this property evolves with material dimensionality is fundamental for exploring its physical characteristics and device applications.

### 2.1. Origin of Ferroelectricity

**Figure 1:**
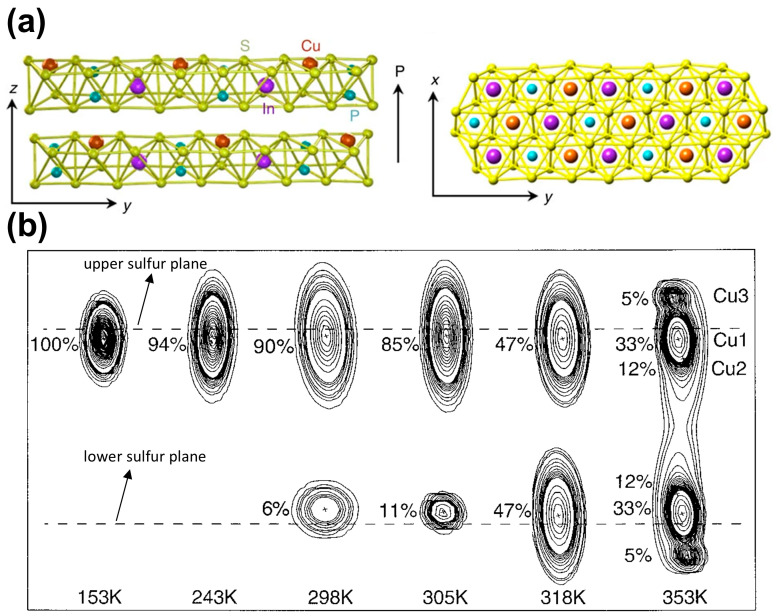
Crystal structure and temperature-dependent copper ion occupancy in CIPS. (**a**) The side view for the crystal structure of CIPS with vdW gap between the layers. Within a layer, the Cu, In, and P–P form separate triangular networks. The polarization direction is indicated by the arrow. Reproduced from Ref. [20]. (**b**) Temperature-dependent evolution of copper ion occupancy at various temperatures (153 K to 353 K). Reproduced from Ref. [52].

At room temperature, bulk CIPS typically possesses a monoclinic crystal structure with the space group Cc. The crystal structure can be described as a sulfur framework, with metal cations (Cu and In) and P–P pairs occupying the octahedral voids, as depicted in Figure 1a. The Cu occupancy determines the orientation of the polarization vector, either downward or upward. Two Cu ion occupation sites near the upper and lower sulfur atoms give rise to upward and downward polarization, respectively. The vdW layers are stacked vdW interactions, enabling them to be readily mechanically exfoliated into nanoflakes with monolayer thickness (~0.72 nm) [51]. Experimental studies have reported that the critical thickness for ferroelectricity in CIPS is around 4 nm [20]. However, whether monolayer CIPS exhibits ferroelectricity remains to be confirmed.

The ferroelectric polarization in CIPS primarily originates from the spatial instability of Cu ions. These ions tend to deviate from the octahedral center, forming local electric dipoles.

As shown in Figure 1b, in the ferroelectric phase (T < T_C_ ≈ 315 K), the Cu ion predominantly occupies a quasi-trigonal site about 1.55 Å above the layer plane (labeled Cu1), establishing a net polarization oriented along the layer normal. This Cu instability arises from the second-order Jahn–Teller effect, where the interaction between the filled 3d^10^ shell and the empty 4s orbital drives monovalent Cu ions away from the high-symmetry center of the sulfur octahedron, favoring lower-coordination, off-center positions. Maisonneuve et al. [52] have provided a detailed elucidation of the thermal evolution of the different copper site occupancies: (i) the off-center, quasi-trigonal Cu1; (ii) the central, octahedral Cu2; and (iii) the nearly tetrahedral Cu3, which extends into the vdW gap. Each of these sites is doubled by a two-fold symmetry axis, creating upward-shifted (Cu^u^) and downward-shifted (Cu^d^) pairs. At low temperatures (e.g., 153 K), the Cu1u site is almost exclusively occupied, identifying the off-center Cu1 position as the ground state of the ferroelectric phase. You et al. [53] and Zhou et al. [54] further found an evident asymmetry distribution of the electronic density around the Cu1 site below the *T_c_*. As temperature increases, the occupancy of Cu1^u^ decreases (e.g., to ~85% at 305 K), while Cu1^d^ becomes populated. This indicates that hopping between these sites begins within the ferroelectric phase. Above the Curie temperature (T_c_), Cu1^u^ and Cu1^d^ become equivalent, restoring a centrosymmetric structure.

### 2.2. Evidence of In-Plane/Out-of-Plane Polarization

Due to its monoclinic crystal symmetry, the ferroelectric polarization in CIPS is not strictly perpendicular to the layer plane. Instead, it possesses a finite tilting angle, which results in the coexistence of both out-of-plane (OOP) and in-plane (IP) polarization components.

For CIPS, polarization characterization via TEM currently encounters fundamental and technical limitations: (1) CIPS is an order–disorder ferroelectric, where its polarization arises from the ordered sublattice arrangement of Cu^+^ ions rather than pronounced lattice distortions [52,55,56] (e.g., oxygen octahedral tilting or central ion displacement) as seen in displacive ferroelectrics, rendering TEM inherently challenging for determining the polarization. (2) Technically, acquiring high-resolution atomic images of CIPS is exceptionally challenging. Unlike perovskite-structured oxides (e.g., PbZr_x_Ti_1-x_O_3_) [57,58,59], CIPS exhibits very blurred atomic imaging—particularly along in-plane [100]crystallographic orientations—and suffers severe electron beam sensitivity [18]. The electron beam irradiation during TEM experiments easily disrupts Cu^+^ ions’ ordering and structural transformation.

**Figure 2:**
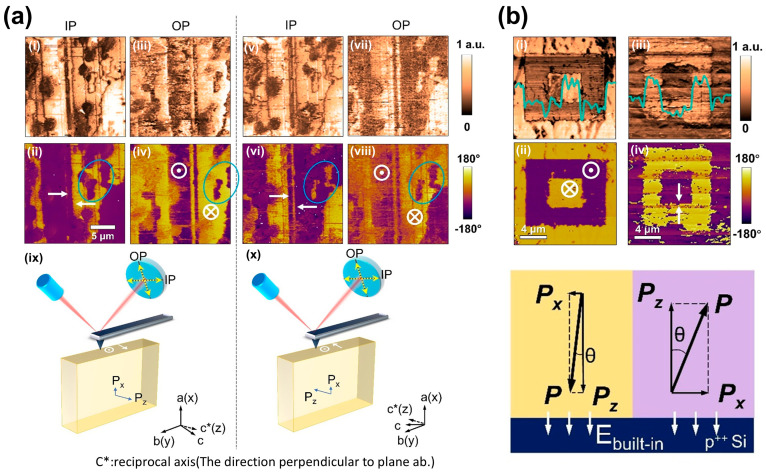
PFM evidence of coexisting IP and OOP polarizations in CIPS. (**a**) Lateral (IP) and vertical (OOP) PFM phase and amplitude images before and after a 180° in-plane rotation of the crystal, with (i)−(iv) before rotation and (v)−(viii) after rotation; (ix) and (x) are illustrations of the vector PFM setup. Only the lateral phase reverses, confirming the inherent IP polarization characteristic of the crystal. (**b**) An enhanced IP piezoresponse in the Pup domain indicates a tilted polarization vector with a finite in-plane component, thus forming a complex polarization domain structure, in which (i) OP amplitude, (ii) OP phase, (iii) IP amplitude, and (iv) IP phase PFM image of a 250-nm-thick CIPS flake with a box-in-box domain pattern. Reproduced from Ref. [60].

In contrast, piezoresponse force microscopy (PFM) is currently the primary approach for resolving the co-existing in-plane (IP) and out-of-plane (OOP) components. Many studies have employed PFM to confirm the coexistence of IP and OOP components in monoclinic CIPS [37,38,60,61]. For instance, Hu et al. [60] provided the evidence for the dipole-locked nature of IP and OOP polarizations. As shown in Figure 2a, after rotating the crystal horizontally by 180°, the phase signal of the lateral PFM image is reversed, whereas the vertical counterpart remains the same. This result confirms the presence of IP polarization in CIPS, instead of the crosstalk artifact from a lateral signal. Figure 2b provides additional confirmation, where the Pup domain exhibits a larger IP piezoelectric amplitude, indicating a tilted polarization vector with a stronger IP component. Together, these findings demonstrate that CIPS possesses a weak but symmetry-allowed IP ferroelectric polarization. Recent years have also witnessed a surge of polar topological textures emerging from the interplay of IP and OOP polarizations in CIPS [37,38]. The co-existing polarizations and competing energies enable the formation of exotic domain structures, such as skyrmion-like nanodomains.

### 2.3. Thickness-Dependent Phase Transition and In-Plane Polarization

**Figure 3:**
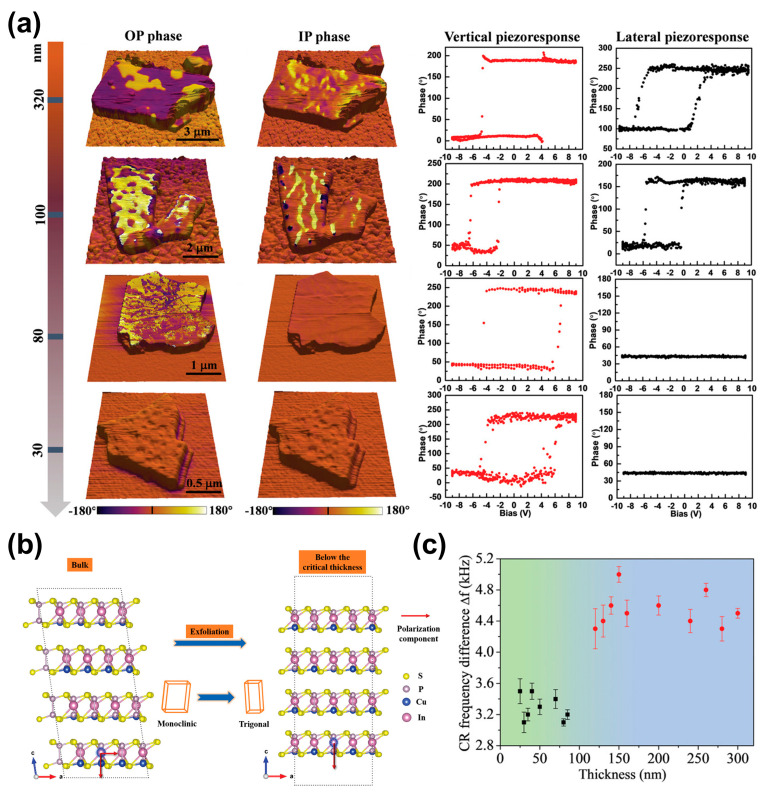
Thickness-dependent ferroelectric and structural evolution in CIPS. (**a**) Thickness-dependent PFM phase images and hysteresis loops showing the disappearance of the in-plane (IP) ferroelectric response below a critical thickness. (**b**) Schematics for the origin of the OP and IP polarization of the bulk CIPS and disappearance of IP polarization in CIPS below the critical thickness. (**c**) The contact resonance (CR) frequency difference between the flakes and silica substrate for CIPS flakes with various thicknesses, showing an abrupt change in the elastic properties of flakes below 90 nm. Reproduced from Ref. [61].

The ferroelectricity of CIPS is highly sensitive to thickness and can be significantly modulated by thickness-dependent structural phase transitions. In bulk form, CIPS generally stabilizes in a monoclinic structure at room temperature with the space group Cc. Due to its low symmetry, this structure allows the spontaneous polarization vector to possess both a prominent out-of-plane (OOP) component and a weaker but distinctly present in-plane (IP) component, which are coupled through the off-center displacement of Cu^+^ ions, exhibiting a “dipole-locking” characteristic. However, when the material thickness is reduced below a certain threshold, a structural phase transition can be induced. Deng et al. [61] revealed that when the thickness of CIPS flakes falls below ~100 nm, the in-plane ferroelectric polarization abruptly disappears, as shown in Figure 3a. Further analysis revealed that near this critical thickness, the crystal structure of CIPS transitions from the monoclinic phase (Cc) to a trigonal phase (P31c), as shown in Figure 3b. Simultaneously, as shown in Figure 3c, the Young’s modulus of the material exhibits a sharp change at this critical thickness, further confirming a thickness-induced phase transition. Nevertheless, recent studies reported that in-plane polarization signals have been observed in CIPS flakes as thin as ~8 nm [38]. A comparative table is presented in Table 1 that summarizes key experimental parameters, including chemical composition, flake thickness, substrate type, crystal structure, OP/IP polarization, and measurement conditions. Based on these results, thickness-driven structural phase transition may be the key factor governing the disappearance of in-plane polarization. This structural transition is likely influenced by multiple factors, such as material quality, Cu content, substrate strain, and interface charge transfer. Herein, we discuss several possible influencing mechanisms, warranting further investigation. Specifically, material quality and composition—especially local Cu-rich regions—may enhance the stability of the monoclinic phase and lower the critical thickness of phase transition. Additionally, interfacial effects such as substrate or composition-induced strain, interface charge transfer, or symmetry coupling could effectively suppress the phase transition and sustain in-plane polarization.

## 3. Chemical Composition Engineering and Emerging Properties

The ferroelectric polarization in CIPS primarily originates from the migration and occupancy of Cu ions within the lattice. Consequently, the modulation of Cu composition has become a crucial approach for manipulating its ferroelectric polarization, domain structure, and novel physical states, providing a unique chemical dimension for understanding and designing functional devices based on CIPS [62,63,64,65,66,67,68,69,70].

### 3.1. Chemical Composition Engineering

The copper content significantly impacts polarization stability [62,64]. Under copper-rich conditions (e.g., at or slightly above the stoichiometric ratio), a uniform, labyrinthine domain morphology is typically favored. However, when the copper content falls below the stoichiometric ratio, this compositional deviation induces phase separation, leading to the spontaneous formation of two chemically distinct phases inside a single crystal: CuInP_2_S_6_ (the ferroelectric phase, CIPS) and a copper-deficient In_4/3_P_2_S_6_ phase (the non-ferroelectric phase, IPS). Consequently, this results in the spontaneous self-assembly of CIPS-IPS heterojunctions at the micro-to-nano scale within the crystal.

**Figure 4:**
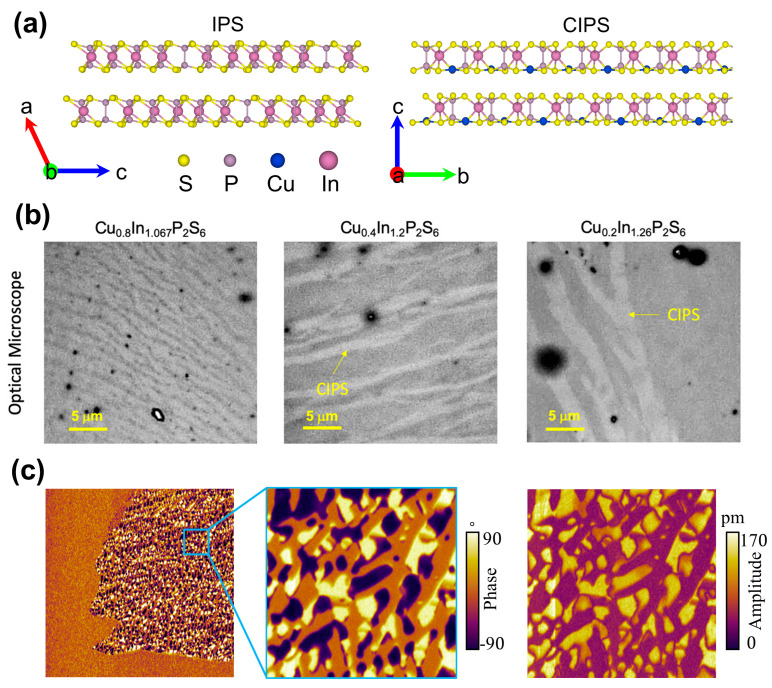
Copper content-dependent phase separation and polarization in CIPS. (**a**) Crystal structures of Cu-deficient In_4/3_P_2_S_6_ (IPS) and ferroelectric CuInP_2_S_6_ (CIPS). Reproduced from Ref. [71]. (**b**) Optical microscopy images showing the coexistence of CIPS and IPS phases under Cu-deficient conditions. Reproduced from Ref. [62]. (**c**) PFM amplitude images revealing embedded non-ferroelectric IPS regions (dark contrast) within the ferroelectric CIPS matrix, forming self-assembled CIPS–IPS heterojunctions. Reproduced from Ref. [71].

Figure 4a,b show the crystal structures of IPS and CIPS, together with the optical microscope images of CIPS-IPS coexisting phases, respectively [62,71]. As shown in Figure 4c, the IPS phases are embedded and coexist with the CIPS phase [71]. In the amplitude image, areas of bright and dark colors represent regions of varying piezoelectric response, with the dark region indicating the paraelectric-phase IPS, where the piezoelectric response is absent. It is noteworthy that the modulation of copper content not only affects domain stability and morphology but also serves as a key role in the formation of multiple polarizations states and topological textures [37,38,60,61]. Recent studies have shown that by controlling the copper content or appropriately substituting Cu, the polarization states and topological textures in CIPS can be effectively manipulated. Xue et al. [38] reported that the appropriate tuning of copper content can stabilize large-area polar skyrmion bubbles through a charge-related energy penalty mechanism. Additionally, chemical doping via partial substitution of Cu^+^ sites with Li^+^ enables the stabilization of the coexistence of high-polarization (HP) and low-polarization (LP) states, thereby laying the foundation for the formation of topological polar structures [72].

It should be noted that our discussion focuses primarily on Cu composition in this review, given that ferroelectricity in CIPS is closely associated with Cu ions. Other stoichiometric and chemical modulations in thiophosphates are also possible. For example, various [P_x_S_y_]^n−^ anionic units (such as PS_3_, P_2_S_6_, and P_2_S_7_) can form depending on synthetic and post-synthetic conditions [73,74]. Although such polyanionic variants have not yet been widely reported in CIPS, their presence may also exert compositional modulation effects on CIPS, warranting further investigation.

### 3.2. Emerging Properties

**Figure 5:**
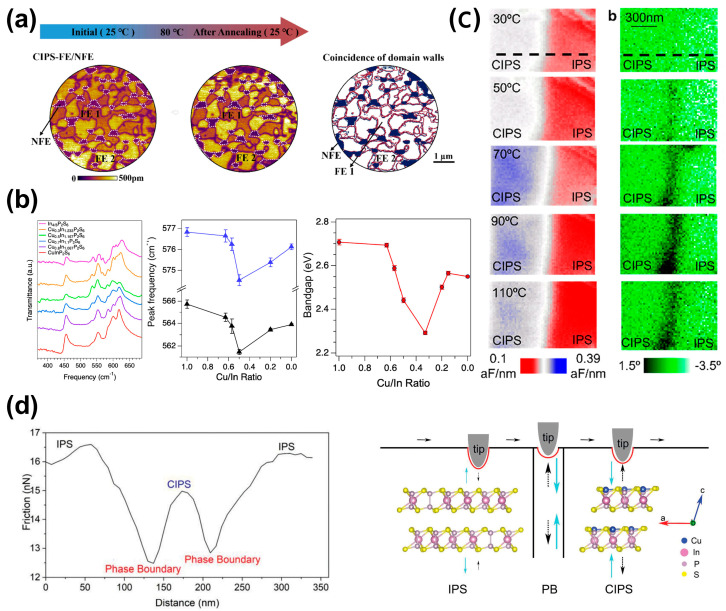
Emergent functionalities enabled by the CIPS-IPS two-phase architecture. (**a**) Thermally robust ferroelectric domains and a shape memory effect induced by strong interfacial pinning at the CIPS/IPS boundary. Reproduced from Ref. [75]. (**b**) FTIR transmission spectra of CIPS-IPS with different Cu contents, and the frequency of the two high-frequency peaks versus the Cu/In ratio, as well as the bandgap versus the Cu/In ratio (obtained from EDS analysis). Reproduced from Ref. [62]. (**c**) Accelerated Cu ion migration along the CIPS-IPS interface, providing a low-barrier pathway for enhanced ionic transport. Reproduced from Ref. [67]. (**d**) Nanoscale friction heterogeneity and reduced friction at the phase boundary arising from lattice mismatch-induced strain. Reproduced from Ref. [76].

The two-phase architecture gives rise to novel physical properties [62,64,65,66,67]. Yang et al. [75] reported the remarkably enhanced thermal stability of ferroelectric domains due to the CIPS/IPS interface. The interfacial region forms a strong “pinning field” and enables exceptional domain stability and a “shape memory” effect after a heating–cooling thermal cycle, as shown in Figure 5a. Rao et al. [62] found that the “reconstruction/chemical pressure” induced by this CIPS–IPS configuration can elevate *T_c_* to approximately 340 K. As shown in Figure 5b, it is further revealed that the lattice strain introduced by the two-phase coexistence modulates phonon behavior and also manifests bandgap tunability [62]. The interfacial strain alters the electronic structure of the material, leading to a significant reduction in the optical bandgap with compositional changes. At a specific composition (e.g.,Cu_0.4_In_1.2_P_2_S_6_, Cu/In ≈ 0.33), the bandgap can decrease to as low as ~2.3 eV, a notable drop compared to the ~2.7 eV of the pure CIPS phase. This provides an additional dimension for tuning in optoelectronic device applications.

Meanwhile, Checa et al. [67] revealed that the chemical potential gradient and lattice distortion present at the CIPS-IPS interface provide a low-barrier pathway for the rapid migration of Cu ions, as shown in Figure 5c. This leads to a significant enhancement of ionic conductivity at the interface, which is key to understanding novel transport phenomena in the system, such as anomalous current rectification and nonreciprocal ferroelectric domain switching.

Beyond their novel electrical responses, the two distinct phases also vary in their mechanical and frictional characteristics [65,71,76]. Zhang et al. [71] confirmed that the Young’s moduli of the CIPS phase and the IPS phase are 27.42 ± 0.05 GPa and 27.51 ± 0.04 GPa, respectively, with the latter being slightly higher than the former. The coexistence of two phases in Cu-deficient CIPS also endows it with novel and tunable friction properties, offering new material insights and theoretical foundations for designing micro-/nano-devices with low friction and high wear resistance. As shown in Figure 5d, Wang et al. [76] reported unusual frictional heterogeneity at the nanoscale, where inhomogeneous friction behavior primarily originates from strain distribution differences caused by a lattice mismatch between the two phases. Due to the largest lattice distortion, the phase boundary exhibits the strongest strain state and ultimately leads to minimized friction. Further studies reveal the wide-range thickness-dependent friction behavior in this two-phase system [65], which goes beyond the previously reported “pinning effect”-dominated mechanism observed only in few-layer materials.

## 4. Ferroionic Coupling and Its Modulation by External Fields

### 4.1. The Concept of Ferroionic Coupling

CIPS stands out among layered vdW ferroelectrics due to its unique polarization mechanism, which is intrinsically linked to the displacement and migration of Cu^+^ ions within the crystal lattice [30,31,52,63,77,78,79,80,81,82,83]. Unlike conventional displacive ferroelectrics, where polarization arises primarily from the relative shift of anions and cations within a rigid framework [84,85], CIPS exhibits long-range ionic migration of Cu^+^ ions across vdW gaps, leading to a distinctive ferroionic coupling that enables richly tunable polarization states.

To further sharpen the scope of ferroionic coupling, we introduce a classification of three regimes based on the degree of Cu ion migration: (i) conventional polarization switching, in which Cu ions are confined within the lattice and typically occur under weak electric fields; (ii) ferroionic coupling, in which Cu ions can migrate over long distances across van der Waals gaps, triggering a series of novel phenomena such as a quadruple-well energy landscape and negative capacitance; and (iii) electrochemical redistribution/reaction, in which extensive Cu ion migration leads to electrochemical redistribution or reaction as the dominant factor, significantly increasing the conductivity and often accompanied by severe degradation or even loss of polarization, typically occurring under strong electric fields.

**Figure 6:**
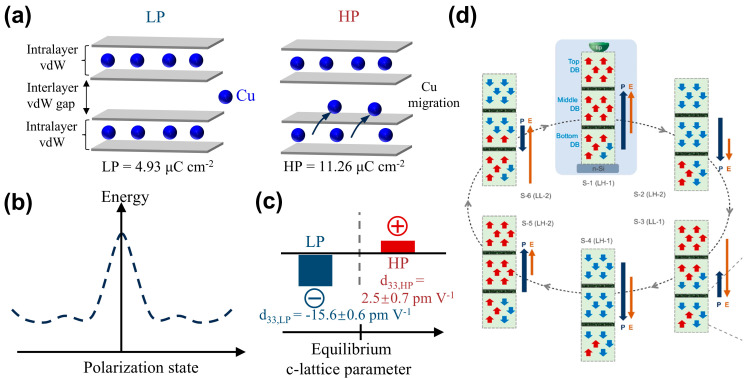
Multistable polarization states and unconventional piezoelectricity in CIPS. (**a**) The relaxation atomic configurations of the LP state and the HP state. Redrawn by the authors based on Ref. [29]. (**b**) Schematic diagram of ferroelectric quadruple-well energy distribution curve. Redrawn by the authors based on Ref. [29]. (**c**) Schematic diagram of piezoelectric response for LP and HP states, Redrawn by the authors based on Ref. [29]. (**d**) Sixfold polarization states realized in CIPS. Reproduced from Ref. [30].

As shown in Figure 6a,b, Brehm et al. [29] revealed in CIPS that Cu ions can not only occupy positions within the vdW layers to form the low-polarization state (LP, ~4.93 µC cm^−2^) but can also partially migrate into the interlayer vdW gaps (interlayer sites), forming a high-polarization state (HP, ~11.26 µC cm^−2^) with comparable energy. This establishes the physical basis for quadruple-well potential: namely, the existence of four energy minima corresponding to the four polarization states, +LP, −LP, +HP, and −HP. Crucially, the transition between LP and HP states via interlayer Cu migration represents the most characteristic evidence of ferroionic coupling, distinguishing it from conventional ferroelectric switching. As shown in Figure 6c, near the equilibrium c-lattice parameter value, the LP state has a large negative piezoelectric coefficient, *d*_333_,_LP_ = −15.6 ± 0.6 pmV^–1^, while the HP state has a relatively small positive piezoelectric constant, *d*_333_,_HP_ = 2.5 ± 0.7 pmV^–1^. As shown in Figure 6d, Li et al. [30] further achieved sextuple polarization states in CIPS, which can be elucidated by the vertically stacked complex antiferroelectric (AFE)/ferroelectric (FE) domain blocks and their interactions with Cu ionic movement crossing the domain.

**Figure 7:**
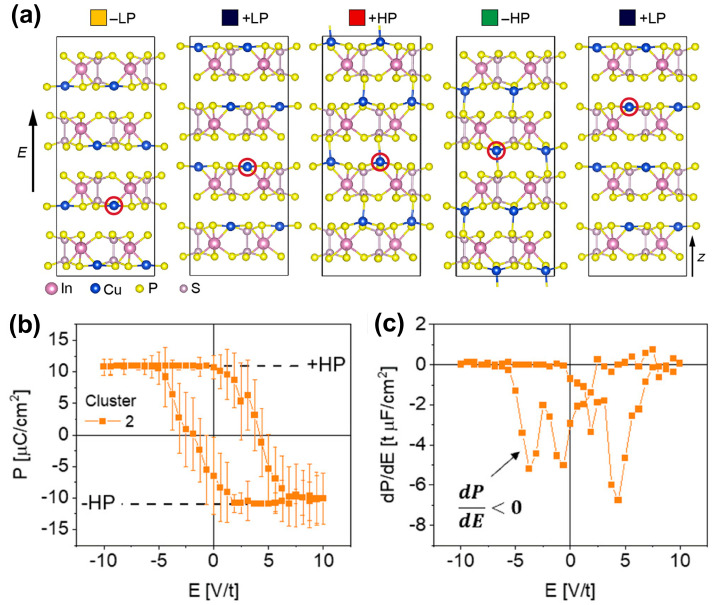
Ferroionic polarization switching and negative capacitance in CIPS. (**a**) Cu ion migration pathways between ±LP and ±HP states, the red circle indicates one specific atom in the structure, which can be moved by the applied electric field. (**b**) Averaged polarization hysteresis loop. (**c**) Derivative for cluster 2. The region of negative slope in the derivative curves is indicated by an arrow. Reproduced from Refs. [31,86].

Beyond its rich polarization states, the distinctive ferroionic coupling in CIPS also leads to novel polarization switching behavior and permanent polarization retention [19,54]. Neumayer et al. [31,86] reported that in CIPS, ferroelectric polarization can align opposite to an applied electric field, offering a novel pathway toward realizing negative capacitance. As shown in Figure 7a, if a sufficiently strong electric field is applied, Cu ions can migrate along the field across the vdW gap to the adjacent vdW layer, causing continuous polarization switching from −LP to +LP, +LP to +HP, +HP to −HP, and then −HP to +LP. This process, in which polarization aligns in a direction opposite to the electric field, will result in a negative slope of polarization versus electric field (dP/dE < 0), satisfying the microscopic condition for negative capacitance, as shown in Figure 7b,c. Fundamentally distinct from quasi-static or transient negative capacitance mechanisms based on Landau theory, this mechanism is rooted in the synergy between ferroelectric order and ionic transport [86], providing a new paradigm for achieving negative capacitance in ferroelectric ionic conductors. Seleznev et al. [25] further identified two distinct polarization switching pathways in CIPS: a cooperative path and a sequential path. Their calculations show that the sequential migration path of Cu ions across the vdW gap has a lower energy barrier. The combination of the two paths leads to a ferroelectric switching cycle embodying the physics of a quantized adiabatic charge pump.

### 4.2. The Experimental Evidence of Cu Ion Migration

**Figure 8:**
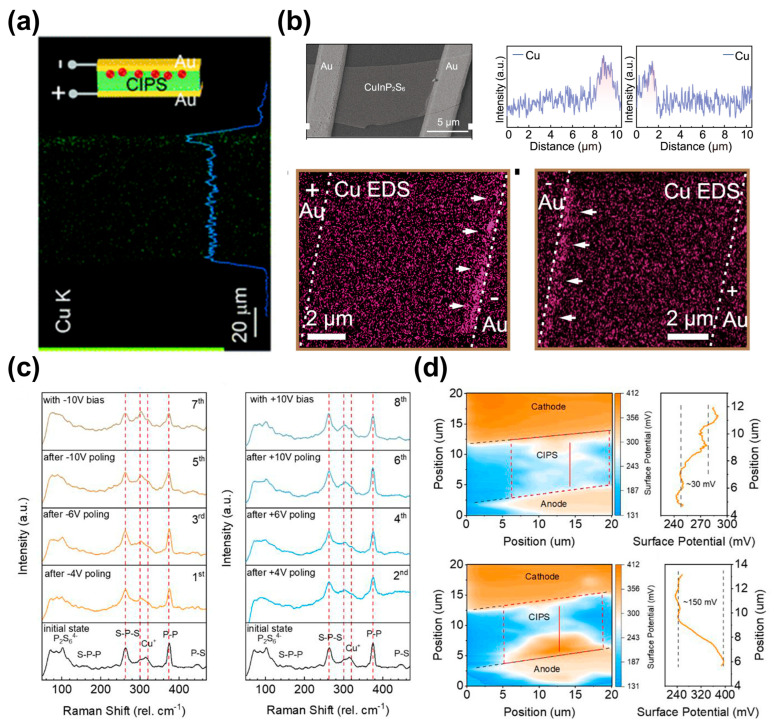
Direct experimental evidence of long-range Cu ion migration in CIPS under electric fields. (**a**) Cross-sectional EDS mapping of a vertical CIPS capacitor after DC stressing. Reproduced from Ref. [54]. (**b**) EDS mapping in lateral devices showing Cu accumulation under bias. Reproduced from Ref. [88]. (**c**,**d**) Raman signatures of bias-induced ionic migration and KPFM potential maps and profiles confirming long-range Cu redistribution. Reproduced from Ref. [89].

Currently, numerous studies have sufficiently demonstrated the long-range Cu ion migration along electric field in both planar and vertical configuration [26,54,87]. Zhou et al. conducted the direct current (DC) stressing tests on a CIPS capacitor with Au electrodes, followed by cross-sectional EDS mapping. As shown in Figure 8a, the results clearly show Cu accumulation at the cathode, confirming the long-range migration and its directionality of Cu ions along the electric field. Zhong et al. [88] presents the evidence of Cu migration in a lateral CIPS homojunction, as shown in Figure 8b. EDS mapping after biasing shows reversible accumulation of Cu near the grounded electrode, while other elements (In, P, S) remain uniformly distributed. Zhu et al. [89] further provides the evidence of Cu ions long-range migration along electric field by Raman and Kelvin probe force microscopy (KPFM), as shown in Figure 8c,d. Guo et al. [18] also offers atomic-scale direct imaging of Cu ion dynamics using aberration-corrected STEM. Along the (100) plane, iDPC-STEM images show Cu ions occupying multiple sites—including lattice, interstitial, and interlayer positions—forming local structures such as Cu_x_InP_2_S_6_ (x = 2–4) under electron beam irradiation. These studies provide the evidence—from element mapping to atomic-resolution imaging—that Cu ions in CIPS are mobile and can migrate over long-range distance under electric fields or external energy.

### 4.3. Polarization Switching Dynamics Under Diverse External Fields

To date, numerous studies have indicated that Cu migration and occupation can be harnessed by diverse external stimuli such as electric fields [19,30,50,83,89,90,91], strain fields [22,81,92,93,94], optical excitation [95,96,97], and other fields [98,99,100,101] (thermal fields, chemical potential fields), leading to richly tunable polarization switching dynamics.

**Figure 9:**
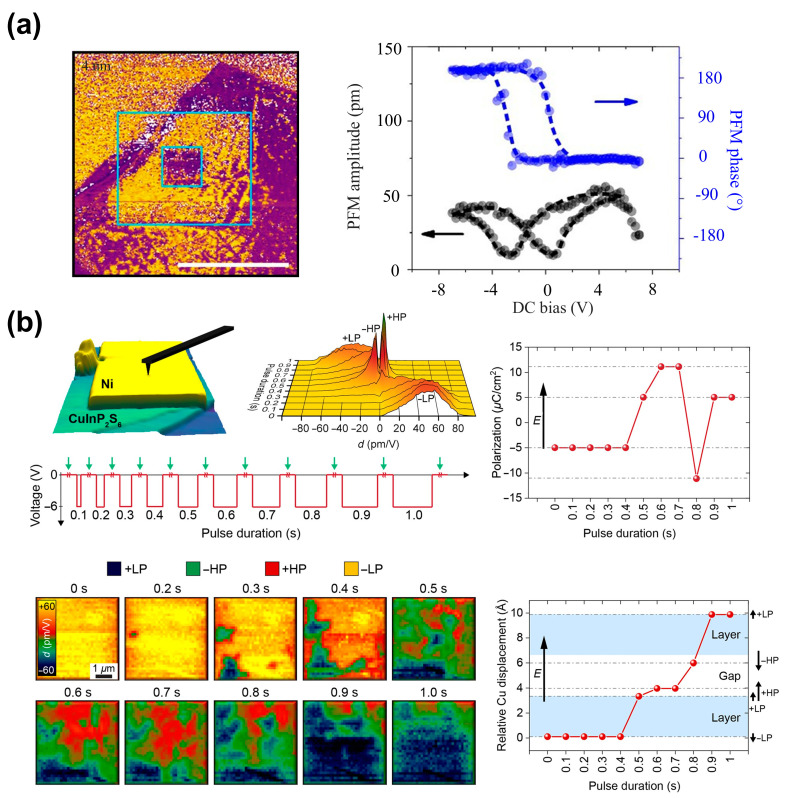
Electric−field−driven tuning of polarization states in CIPS. (**a**) A 4 nm thick CIPS flake with a box-in-box pattern written under reverse DC bias and the corresponding P-E loop curve. Reproduced from Ref. [20]. (**b**) Pulse−width programming of LP/HP state transitions and domain evolution via interlayer Cu migration. Reproduced from Ref. [31].

**Electric field control:** As shown in Figure 9a, Liu et al. [20] applied a reverse *DC* voltage to a 4 nm CIPS flake. The PFM phase image shows out-of-plane polarization switching with clear domain patterns, demonstrating that an external electric field can effectively control the polarization direction. Brehm et al. [29] revealed reversible switching between the +LP and -HP states, as well as between the -LP and +HP states, through the piezoelectric hysteresis loop as a function of *DC* voltage. Through PFM maps, Neumayer et al. [31] further revealed the switching and transitions between different polarization states (LP and HP state) by controlling the duration of the pulsed voltage. 

As shown in Figure 9b, with short pulse durations (0.1–0.7 s), the polarization switches from the negative low-polarization state (−LP) to the positive low-polarization state (+LP) and then to the positive high-polarization state (+HP), aligning with the aplied electric field. However, at a pulse duration of 0.8 s, the polarization switches from +HP to the negative high-polarization state (−HP), where the polarization aligns against the direction of the electric field. Finally, at 0.9 s, the polarization returns to +LP, again aligning with the field. Exploiting this property, Jiang et al. [77] recently reported a cyclic ferroelectric domain manipulation by a unipolar electric field in CIPS, enabled by Cu ion migration across vdW gaps. It further achieved the remarkable “shape memory” effect of manipulated domains and a programmable domain patterning under a unipolar electric field. Liang et al. [50] enabled the configurable kinetic control of cyclic polarization switching and its determined photovoltaic switching via ion migration in CIPS. These results clearly reveal that the electric-field-driven Cu ion migration across vdW gaps is the key origin of multiple polarization states and cyclic domain dynamics.

**Figure 10:**
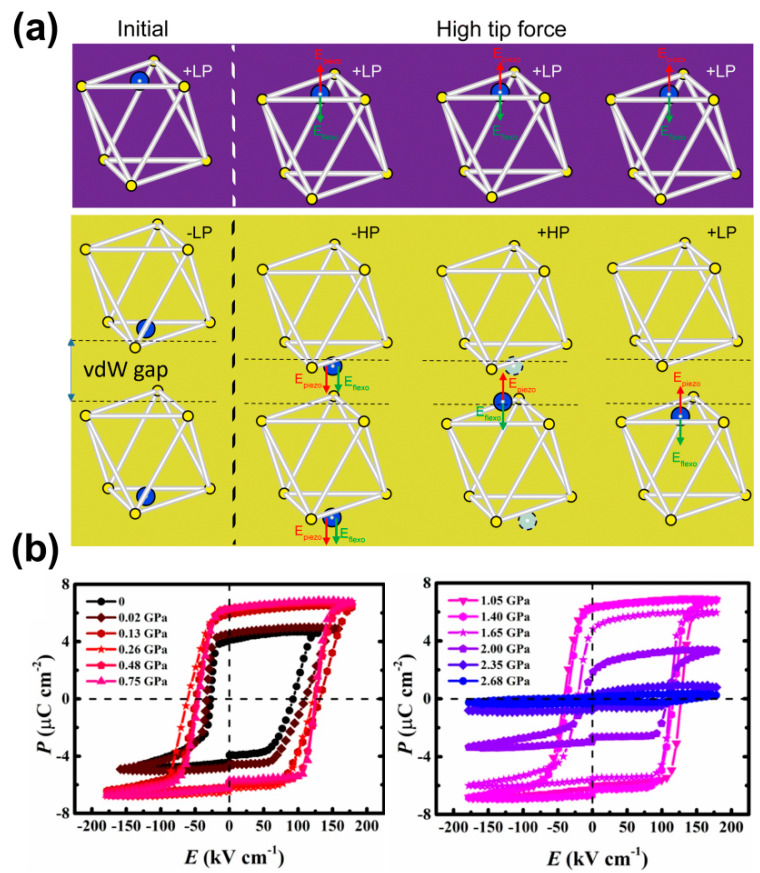
Strain- and force-driven tuning of polar states in CIPS. (**a**) Anomalous reverse mechanical polarization switching in CIPS. Reproduced from Ref. [81]. (**b**) P-E loops of CIPS measured under different pressures. Reproduced from Ref. [33].

**Strain field control:** The energy landscape for Cu displacements is strongly influenced by strain [29]. Compressive strain can compel the movable Cu ions into the vdW gap, thereby actively engineering the LP to HP polar phase. The strain-stabilized HP phase facilitates the formation of polar topological domain structures [37], analogous to the effects achieved through chemical composition engineering [38,72]. As shown in Figure 10a, Wang et al. [81] reported reverse mechanical polarization switching in CIPS nanoflakes, due to the competition between piezoelectric and flexoelectric fields induced by tip pressure, together with the unique quadruple-well state. As shown in Figure 10b, Yao et al. [33] reported a significant polarization enhancement under hydrostatic pressure between 0.26 and 1.40 GPa. Comprehensive analysis suggests that the pressure forces Cu cations to largely occupy the interlayer sites, causing spontaneous polarization to increase. However, under high pressure, the migration of Cu cations to the center of the S octahedron decreases the polarization.

**Figure 11:**
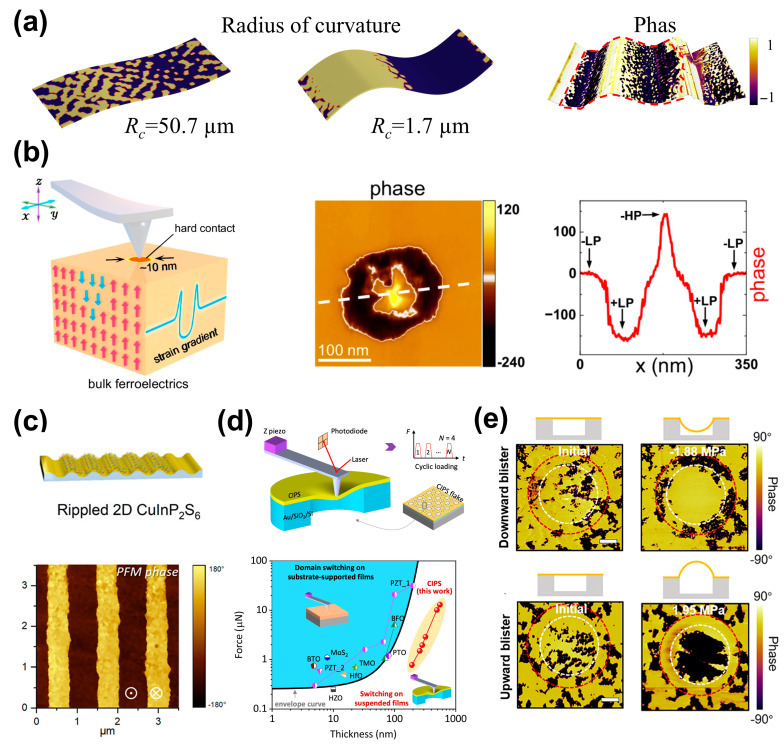
Flexoelectric domain engineering in CIPS enabled by strain gradients. (**a**) Phase-field simulation of polarization distribution under different radii of curvature. Reproduced from Ref. [22]. (**b**) Tip-imprinted strain gradients driving reversible local domain control and stabilizing distinct polarization states. Reproduced from Ref. [102]. (**c**) Substrate-assisted periodic wrinkling producing macroscopic strain-gradient fields and large-area regular domain arrays. Reproduced from Ref. [104]. (**d**) Transverse flexoelectric fields enabling sizable-area domain switching in suspended CIPS nanoflakes over an extended thickness window. Reproduced from Ref. [93]. (**e**) Blister-induced curvature for reversible large-area control. Reproduced from Ref. [92].

Notably, beyond uniform strain fields, non-uniform strain (i.e., strain gradients) also offers a powerful pathway to influence Cu occupancy in CIPS through the flexoelectric effect [22,102,103,104,105,106]. The strain gradient induces a built-in flexoelectric field, which acts on the mobile Cu ions, influencing their migration energy and stable positions. For instance, Ming et al. [22] demonstrated the flexoelectric engineering of ferroelectric domains by designing CIPS with controlled buckled geometries, as shown in Figure 11a. Yang et al. [82] demonstrated that the flexoelectric effect induced by a nanometer tip can effectively reduce the energy barrier for ferroelectric domain switching driven by Cu ion migration. Liu et al. [102] demonstrated the tip-flexoelectric control over polarization states and domain structures, as shown in Figure 11b. The tip imprinting creates a localized strain gradient, leading to the formation of a ring-shaped domain and reversible domain control. This strong field is enough to not only switch polarization but also drive significant interlayer Cu ion migration and the transition from LP state to HP state.

Recent years have witnessed great efforts to achieve large-scale ferroelectric domain engineering beyond the tip approach and have also made significant progress. Chen et al. [104] introduced a designable periodic wrinkle structures into attached CIPS flakes by pre-stretching and releasing a flexible substrate (e.g., PDMS), as shown in Figure 11c. This macroscopic bending creates a uniform strain gradient field, artificially generating large-area, regular ferroelectric domain arrays. Lun et al. [93] realized sizable-area domain switching in suspended CIPS nanoflakes by an introduced transverse flexoelectric field, as shown in Figure 11d. The film thickness range for domain switching in suspended ferroelectrics is significantly improved by an order of magnitude to hundreds of nanometers, being far beyond the limited range of the substrate-supported ones. Liu et al. [92] further achieved reversible flexoelectric control of ferroelectric domain switching over large-area arrays by generating concave–convex curved surfaces via a suspended gas-pressure blister method, as shown in Figure 11e.

**Figure 12:**
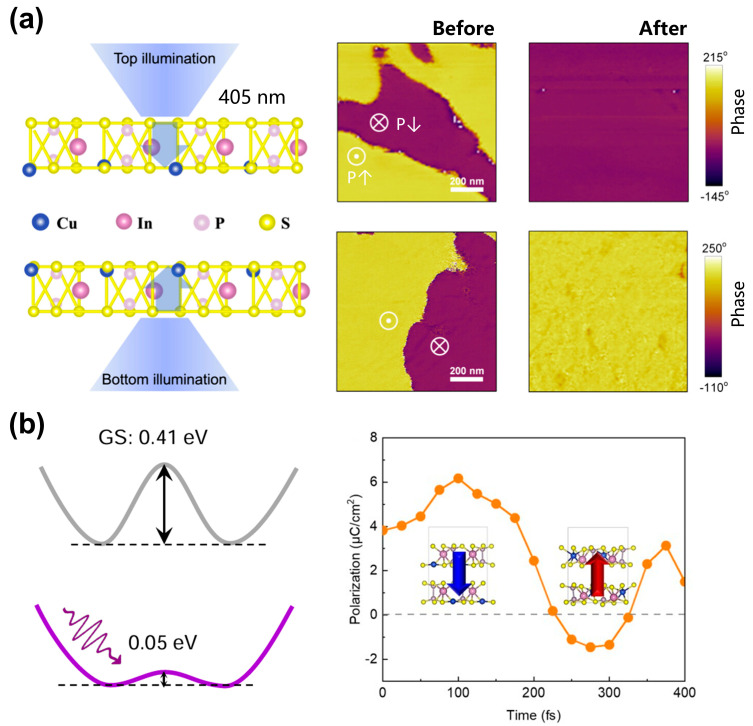
Light-driven ferroionic polarization switching in CIPS. (**a**) Schematic polarization switching via top and bottom illumination and comparison of piezoresponse force microscopy (PFM) Phase mapping under different illumination conditions. Reproduced from Ref. [107]. (**b**) Schematic profiles of the energy barriers at the ground state (GS) and photoexcited state, respectively. Photoexcitation can effectively lower the barrier of intralayer ionic transport and ultrafast polarization dynamics under optical excitation, showing transient polarization reversal on the femtosecond timescale. Reproduced from Ref. [95].

**Optical field control:** Taking advantage of the fact that its polarization switching is intimately coupled with Cu ion migration, Yu et al. [107] demonstrated that the photoinduced deterministic polarization switching in CIPS can be deterministically driven by both above- and below-bandgap illumination via the photothermal effect, as shown in Figure 12a. The optical field can also couple with the built-in electric field generated by Cu ions. For example, Haje et al. [108] reported an anomalous refractive index modulation and giant birefringence in CIPS, revealing a new mechanism of coupling between light and ferroelectric ionic motion. It was also revealed that photo-induced doping significantly reduces the migration energy barrier of Cu ions. For instance, Zhang et al. [95] uncovered ultrafast polarization switching via a laser-activated lower ionic migration barrier, as shown in Figure 12b.

**Thermal field control:** The thermal evolution of different Cu site occupancies has also been investigated in depth, where temperature influences the atomic positions and ionic dynamics. [52,53,54]. Based on the thermal modulation of polarization, Niu et al. [109] demonstrated the pyroelectric response in CIPS via temperature-dependent surface potential changes and realized the CIPS-based pyroelectric nanogenerator. Brehm et al. further revealed the temperature-dependent evolution of quadruple polarization states in CIPS. From room temperature to 55 °C, all four polarization states exist. At 60 °C, the number of polarization states is reduced to three as the −LP state disappears. A further increase in temperature to 65 °C reduces the number of polarization states to one, before the piezoelectric constant of CIPS is indistinguishable from IPS above the Curie temperature. Transition of the polarization states prevents direct measurement of the Curie temperature for HP states, but the evolution of polarization states could be useful for pyroelectric or electrocaloric responses.

**Figure 13:**
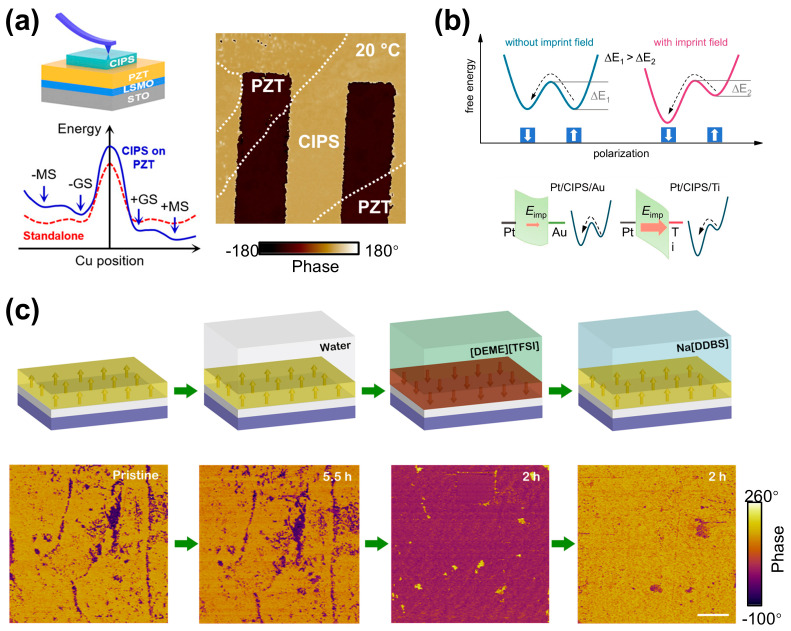
Chemical-potential fields for controlling Cu occupation and polarization states in CIPS. (**a**) Interfacial coupling to a ferroelectric substrate tunes the Cu-position energy landscape and stabilizes distinct polarization states. Reproduced from Ref. [113]. (**b**) Imprint-field engineering using asymmetric metal contacts. Reproduced from Ref. [94]. (**c**) Schematic diagrams and corresponding PFM phase changes in the same CIPS nanoflake after exposure to water, ionic liquid [DEME][TFSI], and Na[DDBS] solution, respectively. Reproduced from Ref. [98].

**Chemical field control:** The Cu occupation and polarization states in CIPS can be also effectively modulated through a chemical potential field such as interfacial charge and chemical doping engineering [54,79,110,111,112]. Wang et al. [113] reported the interface-tuning of ferroelectricity and quadruple-well state in CIPS via a ferroelectric substrate, as shown in Figure 13a. Similarly, Neumayer et al. [114] reported the impact of CIPS–metal (Ag, Cu) interfaces on the stabilization of polar phases and polarization switching, where Cu electrodes tend to stabilize Cu ions in the vdW gap (HP phase) and antiferroelectric states, while Ag enhances piezoresponse.

As shown in Figure 13b, Zhang et al. [94] further revealed that the imprint field introduced through an asymmetric metal contact design (Pt, Ti, Au) with distinct work functions can effectively lower the polarization switching barrier. In addition to these solid–solid interfaces, Xu et al. [98] also demonstrated that the adsorption of organic ions (e.g., [DEME]^+^ or [DDBS]^−^) at CIPS–liquid interfaces can also create surface charge fields that influence Cu ion occupation and reversibly switch polarization, as shown in Figure 13c. Chemical doping, such as with Li^+^, and the modulation of Cu content can also effectively tune the occupancy and distribution of Cu ions, thereby altering the proportion between the low-polarization (LP) and high-polarization (HP) phases [38,72]. This regulation further promotes the formation of topological polar textures, such as polarization bubbles. Overall, these chemical potential fields—from ferroelectric substrate, metal interfaces, dopants, adsorbed ions, chemical doping, or Cu content—provide powerful means to control Cu positioning and polarization states in CIPS, enabling customized functionalities.

## 5. Dynamic Current Response and Conductive Mechanism

Due to its “ferroionic” nature, CIPS combines switchable ferroelectric polarization with highly mobile Cu ions. The polarization switching is accompanied by substantial ionic transport, leading to remarkable conductance modulation. However, the electrical behavior of CIPS is better understood as a field- and timescale-dependent crossover rather than as a set of completely isolated mechanisms. Ferroelectric order and Cu ion migration remain coupled throughout, but their relative weights evolve with bias amplitude and duration. At relatively low fields, the conductance can be mainly polarization-controlled, whereas under stronger bias the contribution from Cu ions’ migration becomes increasingly important and may eventually dominate the current response. The polarization itself in CIPS can modulate the energy barrier for carrier transport at heterojunction interfaces and thus achieve non-volatile conductance states, where the high- and low-resistance states correspond to the two stable polarization orientations [19,20,50,54,100,112].

**Figure 14:**
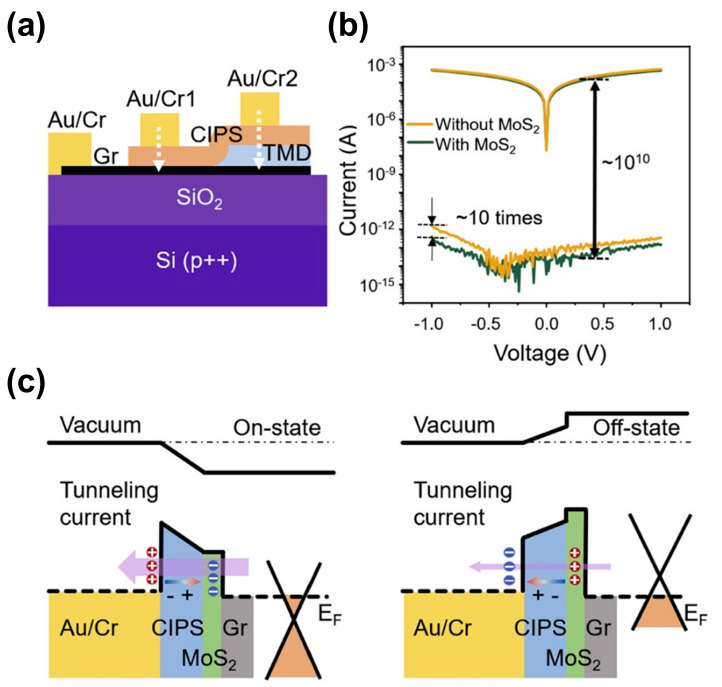
Ferroelectric-barrier-controlled tunneling electroresistance in a Cr/CIPS/MoS_2_/graphene vdW tunnel junction. (**a**) Schematic diagram of the device. (**b**) Current–voltage characteristics of the tunnel junction. (**c**) Band-alignment schematics for the on and off states. Reproduced from Ref. [116].

A representative example of this mechanism is demonstrated in this work by Guo et al. [115], where CIPS serves as the ferroelectric barrier, with graphene and chromium employed as asymmetric contact electrodes. The ferroelectric field effect in CIPS induces a substantial barrier height modulation of up to 1 eV at the junction, resulting in a remarkably high tunneling electroresistance ratio exceeding 10^7^. Subsequently, Wang et al [116]. inserted a monolayer of MoS_2_ between CIPS and graphene, achieving a TER enhancement to over 10^10^. This improvement is attributed to MoS_2_ becoming more insulating in the off state, which simultaneously increases the effective barrier width and height, thereby more effectively blocking electron tunneling, as shown in Figure 14a–c. In this case, the transport behavior is a polarization-controlled conductance modulation.

**Figure 15:**
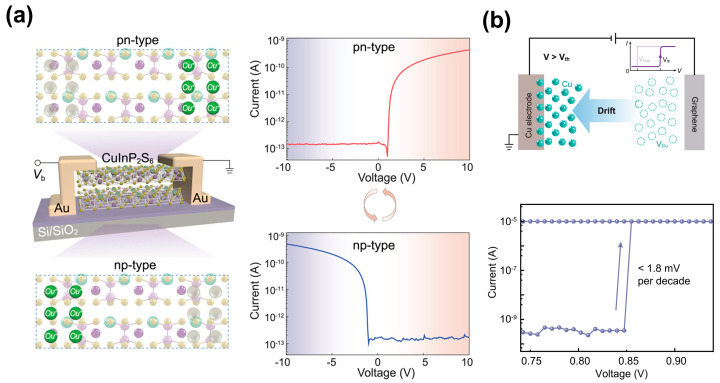
High-field Cu ion migration-dominated conductance modulation in CIPS. (**a**) Reconfigurable pn/np junction formation via Cu concentration gradients. Reproduced from Ref. [88]. (**b**) Ionic-migration-driven threshold switching and conductance jump above Vth. Reproduced from Ref. [118].

Under higher voltages, the conductance modulation mechanism in CIPS shifts from ferroelectric polarization switching to a more dominant Cu ion migration effect [21,23,39,43,117]. The stronger fields drive substantial long-range Cu ion migration, and this migration effect has been extensively explored for understanding dynamic conductive behavior in CIPS. For instance, Zhong et al. [88,118] demonstrated that the Cu migration can also create a gradient in Cu concentration, forming a pn or np junction inside CIPS with regions of a Cu-rich (n-type) and Cu-deficient (p-type) characteristic [88], as shown in Figure 15a. Above a critical voltage threshold, field-driven Cu ion migration in CIPS induces an insulating-to-conducting phase transition, leading to dramatic conductance changes and enabling robust threshold switching behavior [118], as shown in Figure 15b. Overall, in both scenarios described above (threshold switching and homojunctions), the large-field-driven long-range migration of Cu ions disrupts the local ferroelectric order. Their spatial redistribution along the direction of the electric field completely dictates the conductive behavior. Therefore, such responses are better described as ionic redistribution dominating transport.

**Figure 16:**
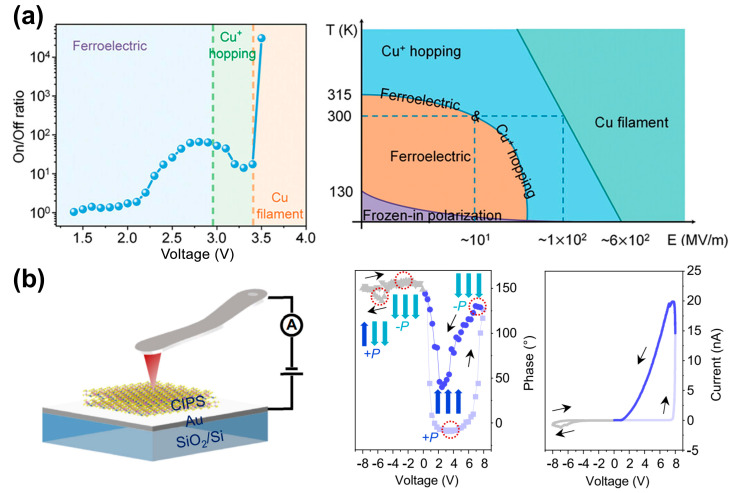
Conductive-mechanism switching in CIPS ferroionic systems. (**a**) Evolution of the ON/OFF ratio with bias voltage and the corresponding temperature–electric-field phase diagram. Reproduced from Ref. [117]. (**b**) Electric-field tailoring of Cu ion migration pathways (intralayer to long-range interlayer) and the associated polarization/transport signatures. Reproduced from Ref. [19].

The conductive mechanism in CIPS involves ferroelectric switching, ion migration, and a corresponding intercoupling, which are highly sensitive to an external electric field [15,24,116,118,119,120]. Distinguishing the dominant contribution of either ferroelectric switching or ion migration to dynamic conductivity remains a challenge. As shown in Figure 16a, Zhou et al. [117] revealed the conducting mechanism transition from ferroelectric polarization to Cu ion hopping to the formation of a conductive filament with an increased bias voltage. This work further obtained the phase diagram of the conductive mechanism transitions at different temperatures and electric fields, providing an in-depth understanding of the complex conductive switching in CIPS.

Jiang et al. [19] further demonstrated that the Cu ion migration pathways determines the conductive mechanism by tailoring the electric fields, as shown in Figure 16b. As the magnitude or duration of the positive bias increases, Cu ion migration gradually shifts from intralayer to long-range interlayer pathways. This shift is accompanied by the onset of polarization alignment against the electric field as evidenced by the polarization switching hysteresis loop, which favors a transition from a ferroelectric to ion migration-dominated conductance mechanism and an abrupt current increase. These works largely deepen the understanding of ion migration dynamics and conductive mechanism switching in CIPS ferroionic systems.

## 6. Emerging Nanoelectronics Based on CIPS Ferroelectrics

In CIPS, ferroelectricity and ion migration provide a novel platform for developing new types of low-power, high-integration, and multifunctional memory, logic, and computing chips [121,122,123,124,125,126,127,128,129]. This section primarily focuses on advances in core application areas, such as nonvolatile memories, optoelectronics, and neuromorphic computing. For clarity, the representative types of CIPS-based devices discussed in this section and their governing mechanisms are summarized in Table 2.

**Figure 17:**
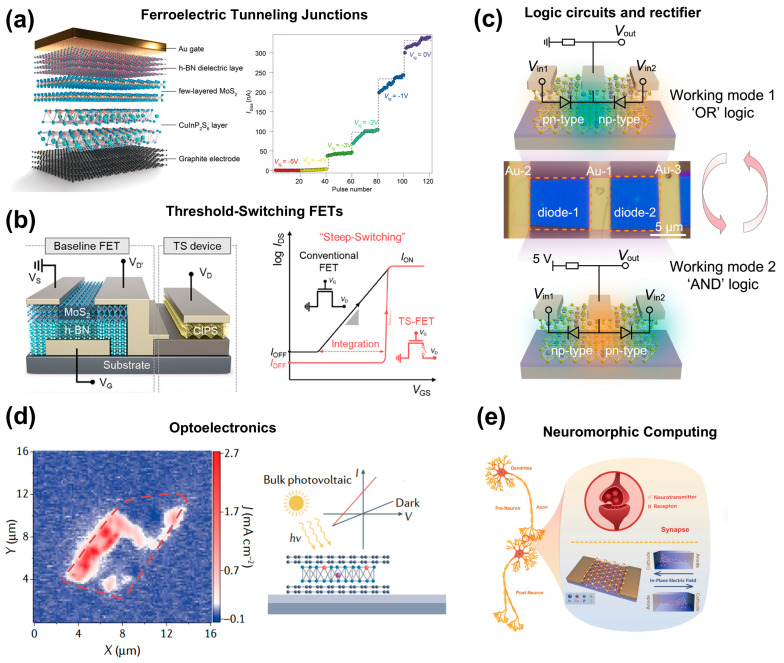
Device concepts and applications enabled by ferroionic Cu dynamics in CIPS. (**a**) Three-terminal gate-programmable vertical memory based on a CIPS heterojunction. Reproduced from Ref. [42]. (**b**) Threshold switching FETs realized by integrating a CIPS threshold switching unit with a channel FET, enabling steep-slope switching. Reproduced from Ref. [133]. (**c**) Cu-migration-programmable homojunction devices for self-rectification and reconfigurable logic. Reproduced from Ref. [88]. (**d**) Ferroelectric/ionic-dynamics-enabled optoelectronics in CIPS. Reproduced from Ref. [134]. (**e**) Neuromorphic and optoelectronic synapses based on CIPS memristive switching. Reproduced from Ref. [14].

**Nonvolatile memories.** A ferroelectric tunnel junction (FTJ) is a functional device that modulates tunneling resistance by leveraging the polarization direction of the ferroelectric barrier layer, making it a promising candidate for future low-power, non-volatile memory and computing-in-memory applications. Currently, significant progress has been made based on the CIPS ferroelectric system [130,131,132]. Wu et al. [115] reported a breakthrough in the Cr/CIPS/graphene vdW ferroelectric tunnel junction (vdW FTJ). This architecture demonstrated a record-high tunneling electroresistance (>10^7^), which originates from the substantial Fermi level shift in graphene modulated by the adjacent CIPS layer. The all-vdW nature of the CIPS/graphene interface facilitates a giant tunnel barrier height modulation of ~1 eV, significantly exceeding that of conventional perovskite oxide ferroelectrics and fluorite ferroelectrics. As shown in Figure 17a, Li et al. [42] further fabricated a three-terminal, gate-programmable vdW vertical heterojunction memory with a structure of graphite/CIPS/MoS_2_/h-BN gate dielectric. This architecture integrates the functionalities of both a ferroelectric memristor and a MOS field-effect transistor into a single device. The ferroelectric memristive characteristics of the device can be enabled/disabled and multi-level tuned via the top gate, achieving the programming of the memory function.

By integrating CIPS with field-effect transistors (FETs), a threshold switching FET (TS-FET) with steep switching characteristics can be constructed. This is a key technology for realizing ultra-low-power logic and neuromorphic hardware. As shown in Figure 17b, Baek et al. [133] fabricated a TS-FET by connecting a CIPS threshold switching unit in series with an MoS_2_ channel FET. The device achieved outstanding performance metrics: a steep subthreshold swing of 7.5 mV/dec, a high on/off current ratio (>10^7^), and an ultra-low off current (≈0.3 pA). The underlying mechanism involves rapid Cu ion migration and a transition of the CIPS layer to a low-resistance state when the applied voltage reaches the threshold, which triggers the voltage to be rapidly redistributed to the MoS_2_ channel and induces an abrupt current. Beyond serving as an ultra-steep switch, dynamic ion migration can be effectively applied in self-rectifier and programmable logic circuits. For instance, Zhong et al. [88] developed a two-terminal reconfigurable homojunction device based on CIPS, as shown in Figure 17c. The device operates by the migration of Cu ions under an applied electric field, which creates a tunable pn- or np-type junction along the channel. This enables a high rectification ratio of up to 10^4^, a low leakage current of ~100 fA, and a high breakdown voltage of 170 V. The device also demonstrates versatile applications in reconfigurable electronics, including signal-processing rectifiers operating at frequencies up to 10 kHz and programmable logic circuits (such as OR and logic) without altering input connections.

**Optoelectronic devices.** CIPS has emerged as a particularly compelling candidate for advanced optoelectronic applications, owing to its exceptional dynamic tunability. Its innate ionic dynamics and ferroelectricity provide a unique physical basis for creating optoelectronic devices. As shown in Figure 17d, by its inherent ferroelectric polarization, Li et al. [134] confirmed a bulk photovoltaic effect in CIPS with an enhanced photocurrent density that is two orders of magnitude higher than in conventional bulk perovskite ferroelectrics, showing potential for solar cell applications. By constructing asymmetric heterojunctions (such as Pt/CIPS/graphene), this photovoltaic effect can be amplified programmably [135]. Ion migration further offers an additional knob to control the photovoltaics. Liang et al. [50] reported that Cu ion migration in CIPS can achieve configurable kinetics of polarization switching and its determined photovoltaic response. Zhong et al. [88] revealed a reconfigurable photovoltaic performance based on the homojunction formed via Cu ion migration. Unlike fabricated p-n junctions fixed during manufacturing, the polarity and barrier height of a CIPS homojunction can be electrically programmed and erased, enabling a reconfigurable optoelectronic response.

**Neuromorphic computing.** The kinetic processes of ion migration in CIPS—such as relaxation time and conductivity thresholds—closely resemble the weight-updating characteristics of biological synapses, making it a natural platform for emulating neural synapses and complex neuronal networks [43,45,136,137,138,139,140,141]. Its programmable conductance states can function as synaptic weights within neural networks. As shown in Figure 17e, Chen et al. [14] demonstrated that a lateral two-terminal memristor based on CIPS, through electric-field-modulated Cu ion migration, successfully emulates various synaptic behaviors, including short-term plasticity (STP), long-term plasticity (LTP), and spike-timing-dependent plasticity (STDP), as well as advanced neural functions such as Pavlovian conditioning and activity-dependent synaptic plasticity (ADSP). Sun et al. [26] reported a memristor based on ionic CIPS, in which up to 1350 linear conductance states are achieved by controlling Cu ion migration. In addition, the device shows a low operation current of 100 pA. Complex functions such as signal transport from one neuron to multiple neurons or multiple neurons to one neuron are achieved by CIPS-based device arrays. Recently, optoelectronic synapses based on CIPS have been further developed for artificial visual system applications [106,134,136,142,143,144,145,146,147,148,149,150,151]. Liu et al. [136] fabricated graphene/CIPS/Au optoelectronic synapses and achieved a low energy consumption (~3.32 fJ per event), making them suitable for scalable integration in artificial visual systems. Men et al. [135] further achieved a tenfold enhancement of the photocurrent through polarization modulation. The device can be applied to in-sensor computing and has demonstrated outstanding performance in tasks such as image edge detection and pattern classification. It is worth noting that although recent years have witnessed significant progress in this field, such cutting-edge research remains in lab, with a considerable distance yet to be bridged before industrial applications can be realized.

**Table 2:** Representative types of CIPS-based devices and their governing mechanisms.

Device Structure	Type	Reference	Dominant Mechanism
Cr/CIPS/graphene vdW	Ferroelectric tunnel junction	Wu et al. [115]	Ferroelectric switching
graphite/CIPS/MoS_2_/h-BN	Three-terminal memory	Li et al. [42]	Ferroelectric switching
graphite/CIPS/graphite	Bulk photovoltaic device	Li et al. [134]	Ferroelectric switching
graphite/CIPS/graphite	Photovoltaic device	Liang et al. [50]	Strong ferroelectric-ionic coupling
Pt/CIPS/Gr	Optoelectronic synaptic device	Men et al. [135]	Strong ferroelectric-ionic coupling
Au/CIPS/Au	Artificial synapse	Ci et al. [39]	Strong ferroelectric-ionic coupling
a MoS2 FET + a CIPS TS	Threshold switching transistor	Baek et al. [133]	Ion migration-dominated
Au/CIPS/Au	Reconfigurable logic device	Zhong et al. [88]	Ion migration-dominated
Cr/CIPS/Au	Lateral two-terminal CIPS memristor	Chen et al. [14]	Ion migration-dominated
graphite/CIPS/graphite	Ionic CIPS memristor	Sun et al. [26]	Ion migration-dominated
graphene/CIPS/Au	Optoelectronic synapse	Liu et al. [136]	Ion migration-dominated

## 7. Challenges and Outlook

While CIPS offers a unique material platform and physical foundation for developing high-performance memory and neuromorphic computing devices, there are several points that are worth paying attention to. From the perspective of ferroelectricity, ferroelectricity has been confirmed in nanoflakes down to ~4 nm [20]. However, whether it exists in thinner or even monolayer CIPS remains an open question. Clarifying this issue is essential for ultra-scaled device integration in the post-Moore era. Additionally, compared to the out-of-plane direction, the in-plane ferroelectric polarization of CIPS remains largely unexplored, with only a few recent studies beginning to address this topic [60,61]. In-plane polarization is vital for enabling high-density memory architectures and the formation of topological polarization textures. How the Cu ion and its migration affects in-plane polarization ordering and switching dynamics warrants further investigation. From a perspective of nanoflake preparation, the current synthesis of CIPS nanoflakes relies on chemical vapor transport growth of bulk crystals followed by mechanical exfoliation [17,28,109,152,153,154,155]. While some progress has been made in producing CIPS nanosheets via intercalation and exfoliation methods [16,156], the preparation of wafer-scale, uniform, and high-quality CIPS films remains a significant challenge. Developing reliable large-area synthesis techniques is crucial for practical applications. From a perspective of device applications, although the dual role of Cu ions endows CIPS-based devices with numerous unique properties, yet it also raises concerns regarding device stability and endurance due to Cu ions’ migration under sustained electrical operation [49]. Systematic studies on the long-term reliability, cycling endurance, and ion-migration control in CIPS-based devices are urgently needed to ensure their operational robustness. Addressing these challenges will not only deepen the fundamental understanding of CIPS but also accelerate its integration into functional memory, logic, and neuromorphic systems. Great efforts in material synthesis, device engineering, and physical mechanism will be key to unlocking the full potential of this versatile ferroionic CIPS material.

## Figures and Tables

**Table 1:** Comparison of composition, thickness, substrate, OP/IP, and measurement conditions for representative studies on the critical thickness limit of in-plane polarization in CIPS.

Composition	Thickness	Substrate	Structure	OP	IP	Measurement	Reference
−	>100 nm	heavily doped silicon	Monoclinic Cc (No. 9)	√	√	Vector-PFM	[61]
−	<100 nm	heavily doped silicon	Trigonal (P31c)	√	×	Vector-PFM	[61]
Cu_0.9_In_0.99_P_2_S_5.9_	8–200 nm	Au-covered silicon	Monoclinic Cc (No. 9)	√	√	Vector-PFM	[38]

## Data Availability

No new data were created or analyzed in this study. Data sharing is not applicable to this article.
